# A Comparative Study of Ranibizumab and Aflibercept for Neovascular Age-Related Macular Degeneration: 12-Month Outcomes of Polish Therapeutic Program in Non-Tertiary Institution

**DOI:** 10.7759/cureus.15916

**Published:** 2021-06-25

**Authors:** Tomasz Skrzypczak, Aleksandra Jany, Ewa Bugajska-Abramek, Joanna Bogusławska, Agnieszka Kowal-Lange

**Affiliations:** 1 Faculty of Medicine, Wroclaw Medical University, Wroclaw, POL; 2 Department of Ophthalmology, Regional Specialist Hospital in Wroclaw, Research and Development Center, Wroclaw, POL

**Keywords:** amd, ranibizumab, aflibercept, therapeutic program, aflibercept intravitreal injection, ranibizumab intravitreal injection

## Abstract

Introduction

This single-center study aimed to compare the 12-month treatment outcomes of ranibizumab with that of aflibercept in routine clinical practice.

Methods

Cohort of patients diagnosed with treatment-naïve neovascular age-related macular degeneration (AMD), treated using either ranibizumab (n = 33 eyes) or aflibercept (n = 44 eyes) monotherapy over a 12-month follow-up period was analyzed. Anonymous data were extracted from the electronic database dedicated to the drug program.

Results

In the ranibizumab group, there were no statistically significant changes in best-corrected visual acuity (BCVA) (Early Treatment Diabetic Retinopathy Study [ETDRS] letters) and central retina thickness (CRT) (µm), between baseline (67.9 ± 8.6 & 384.9 ± 97.9) and at 12 months (67.9 ± 12.1 & 398.9 ± 127.1; P = 0.372 & P = 0.884, respectively). In the aflibercept, there was an improvement in BCVA and reduction in CRT between baseline (64.2 ± 8.1 & 414.3 ± 97.8) and at 12 months (70.7 ± 7.4 & 342.3 ± 71.6; P < 0.001 & P < 0.001, respectively). There was no difference in BCVA between the two groups at either diagnosis (P = 0.101) or 12 months (P = 0.917). Mean number of injections in the ranibizumab group was significantly lower (4.9 ± 1.5) than in the aflibercept group (6.7 ± 1; P < 0.001).

Conclusions

One initial injection of ranibizumab and then pro re nata (PRN) regimen resulted in stabilization of disease progression. Drug selection and treatment scheme could influence twelve-months outcomes. In the aflibercept group, three initial monthly injections and then every two months provided both significant BCVA improvement and CRT reduction at 12 months of treatment.

## Introduction

Age-related macular degeneration (AMD) is a chronic disease that affects around 50-70 million people worldwide and is the leading cause of blindness in developed countries, including Poland [1]. The treatment of choice for wet AMD is intravitreal injection of vascular endothelial growth factors inhibitors (VEGF) [2-3]. Although anti-VEGF therapy is focused on treating symptoms, it has revolutionized the treatment of exudative AMD [4].

Since November 2015, treatment of patients with wet AMD in Poland has been financed by the National Health Found (NHF). Two drugs, aflibercept or ranibizumab are administrated as part of the Polish National Treatment Program [5]. The authors aimed to compare the effectiveness of ranibizumab and aflibercept, in non-tertiary institution. Internal audit of the therapeutic program outcomes in the authors' institution was the purpose of this study.

The manuscript was posted on pre-print server, Research Square: https://doi.org/10.21203/rs.3.rs-425927/v1.

## Materials and methods

Study design

This was a non-randomized, retrospective, observational single-center study of treatment-naïve eyes for wet-AMD. Anonymous data were collected from the drug program dedicated electronic database. Therapeutic Program Monitoring System (TPMS) is central database, supervised by NHF utilized to monitor execution of the therapeutic program [6]. TPMS records from December 2015 to December 2020 were analyzed. This study was approved by the hospital bioethical committee and conducted in accordance with the tenets of the Declaration of Helsinki.

Data included the assessment of the following: the best-corrected visual acuity (BCVA) on a decimal scale made on the basis of a Snellen chart, macular morphology with automatic measurement of the central retina thickness (CRT) from the central subfield of the optical coherence tomography (OCT); percentage share of active area in degenerative lesion, size of the degenerative lesion (DA). Type of neovascularization was determined with fluorescein angiography (FA). All OCT scans were performed on certified spectral-domain OCT (Spectralis OCT, Heidelberg Engineering, Germany). In addition, possible prior treatment with VEGF inhibitors, disease activity defined as the presence of sub- or intra-retinal fluid in OCT or a new hemorrhage were reported. 

Demographic factors extracted from the database were age (years), gender, eye (right/left), date of diagnosis, dates and number of injections with VEGF inhibitors.

Treatment program

The following inclusion criteria were applied in the treatment program: (1) presence of active (primary degeneration, not secondary) occult (type 1), classic (type 2) or mixed (other than pure classic or occult, including retinal angiomatous proliferation - type 3) Subretinal choroidal neovascularization (CNV) occupying more than 50% of the lesion; (2) age years; (3) total size of degenerative lesion less than 12 optic disc area (DA); (4) BCVA in the treated eye of 0.1-0.8 (from 2015 to 2017); 0.2-0.8 (from 2017 to present), determined on a decimal scale according to the Snellen chart; (5) absence of dominant geographic atrophy or hemorrhage in the whole lesion (more than 50% of the lesion); (6) informed consent of the patient to undergo intravitreal injections. Patients with scarring or atrophy of the fovea were not eligible for the treatment.

BCVA deterioration to according to Snellen’s chart lasting longer than two months excluded patient from the program. In addition, physicians could remove the patient from the program according to the exclusion criteria contained in summary of product characteristics. Only treatment-naive patients, continuously enrolled in the therapeutic program for at least 12 months were included in the study. 

Aflibercept or ranibizumab were chosen by the physician at the time of the first injection. Ranibizumab was mainly administrated in cases that were expected to improve rapidly, allowing for a prompt withdrawal from the therapeutic program. These patients had higher BCVA and lower CRT at baseline than patients qualified for aflibercept regimen. Cut-off values were at the discretion of physician and not known to the authors. All eyes were treated with fixed regimen, according to the “Treatment of neovascular form of age-related macular degeneration” National Health Found program guidelines [6].

Three monthly intravitreal injections of aflibercept (2.0 mg/0.05 mL) were administrated and then the drug was injected every two months. After the first 12 months of aflibercept treatment, a pro re nata (PRN) regimen was in force. In patients treated with ranibizumab, after administering the initial dose (0.5 mg/0.05mL) the PRN scheme was applied. Patients received injections monthly until the disease was no active and functional stability was achieved. Then, patients were followed-up for four to eight weeks. Ranibizumab was given again, when active neovascularization recurred, and functional parameters worsened. Switch from ranibizumab to aflibercept and vice versa was permitted under the conditions described in the program guidelines [6]. However, patients which drug was switched during 12 months of therapy were excluded from the analysis.

Clinical examinations, OCT and measurement of BCVA were performed at each follow-up visit and recorded in the electronic database. Patients with follow-up period $&lt;$ 12 months were excluded from the analysis. Only, patients with treatment-naïve wet AMD eyes and complete medical records were included in the study.

Changes after 12-month treatment

The baseline BCVA and CRT were compared with those at 12 months within each treatment group. 

Comparisons between the ranibizumab group and aflibercept group

Eyes were divided into two groups: the ranibizumab (n = 33) and aflibercept groups (n = 44). These were compared in terms of baseline characteristics (BCVA, CRT, percentage share of active area in degenerative lesion, size of degenerative lesion, neovascularization type, age and sex). In addition, the BCVA, CRT and number of injections after 12 months of treatment were compared between groups. 

To investigate the difference between good visual outcome and poor visual outcome, eyes were divided into two groups on the basis of BCVA at 12 months. These with BCVA $\geq$ 20/50 were assigned to the “good visual outcome” group. Patients with BCVA $&lt;$20/50 were included in the “poor visual outcome” group. Within each treatment group, the visual outcomes were compared in terms of baseline characteristics (mentioned above) and number of injections. 

The patients were divided into two groups, according to the number of injections received within 12-month treatment period. Mean number of injections were calculated for ranibizumab and aflibercept groups. Patients, whose number of injections was $\geq$ than mean in their drug group were compared against these, whose number of injections was $&lt;$ than mean in their drug group. The BCVA and CRT at baseline and at 12 months were compared between these groups. 

For the purpose of the study, visual acuity was calculated from the Snellen decimal scale to the number of ETDRS letters [7]. The CRT values were reported in µm.

Statistics

Statistical analyses were performed with open-source software JASP, version 0.14.1, https://jasp-stats.org. Data were collected with the use of Microsoft Excel software, version 16.46, (Microsoft Corporation, Redmond, WA, USA). The normality of data was examined with Shapiro-Wilk test. Differences between two time points were analyzed using the Wilcoxon signed-rank test. To analyze differences involving parametric values between two groups, either the independent t-test or the Mann-Whitney U test were utilized. Those that concerned non-parametric values were analyzed using the chi-square test. P-values < 0.05 were considered significant. Data were presented as mean ± standard deviation (SD), where applicable. 

## Results

During the analyzed period, 67 patients (77 eyes) met the eligibility criteria. Both eyes of 10 patients (14,9%) were enrolled in the study. The mean age was 80.3 ± 7.4 years. The ranibizumab group consisted of 33 eyes and the aflibercept included 44 eyes (Table 1).

**Table 1:** Baseline patient characteristics (n = 67), 77 eyes. Data presented as mean ± standard deviation where applicable. BCVA: best-corrected visual acuity; ETDRS: Early Treatment Diabetic Retinopathy Study.

Characteristics	
Age, years	80.3 ± 7.4
Sex, no. (%)	
Men	27 (40,3%)
Women	40 (59.9%)
Type of neovascularization, no. (%)	
Classic (type 2)	24 (31,2%)
Occult (type 1)	24 (31.2%)
Mixed	29 (37.6%)
Size of lesion (DA)	2.66 ± 0.02
Surface (%)	60% ± 7%
Central retinal thickness (CRT, µm)	401.7 ± 98.3
Baseline BCVA (ETDRS), at diagnosis	65.9 ± 9.2

Changes in BCVA and CRT

**Figure 1:**
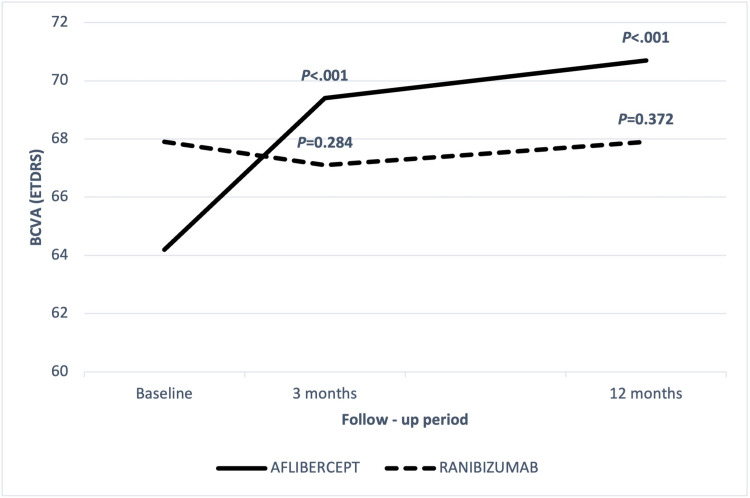
Changes in BCVA in patients treated with ranibizumab or aflibercept. BCVA: best-corrected visual acuity; ETDRS: Early Treatment Diabetic Retinopathy Study.

In the ranibizumab group, the BCVA values at diagnosis, three months and 12 months were 67.9 ± 8.6, 67.1 ± 17.2, 67.9 ± 12.2, respectively (Figure 1).

**Figure 2:**
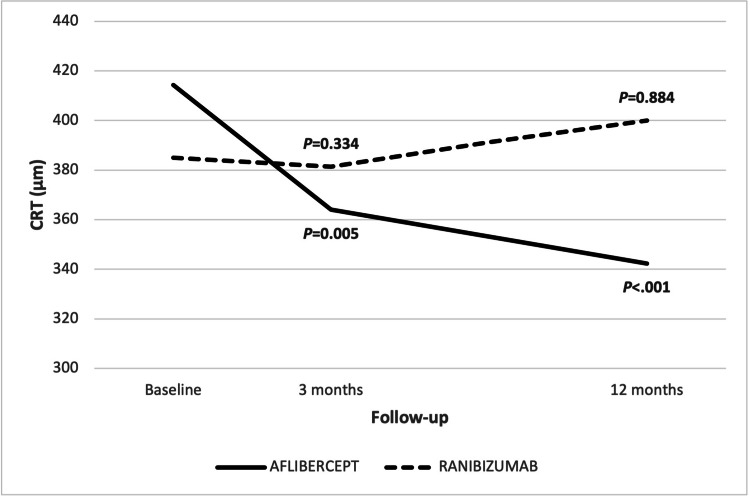
Changes in CRT (µm) in patients treated with ranibizumab or aflibercept. CRT: central retina thickness.

Neither BCVA at three months nor BCVA at 12 months changed significantly from baseline (P = 0.284 & P = 0.372, respectively). There was no significant change in BCVA between three and 12 months (P = 0.687). The CRT values at diagnosis, three months and 12 months were 384.9 ± 97.9, 381.4 ± 119, 400 ± 126.8, respectively (Figure 2). There were no statistically significant differences in CRT values between baseline, three and 12 months (P = 0.334 & P = 0.884, respectively). In addition, the difference between CRT values at 3 and 12 months was not statistically significant. 

In the aflibercept group, the BCVA values at diagnosis, three months, and 12 months were 64.2 ± 8.1, 69.4 ± 8, 70.7 ± 7.4, respectively (Figure 1). The BCVA values at 3 and 12 months significantly improved from baseline (P < 0.001 & P < 0.001). The difference between BCVA values at 3 and 12 months was not significant (P = 0.332). The CRT values at diagnosis, three months and 12 months were 414.3 ± 97.8, 364.02 ± 104, 342.3 ± 71.6, respectively (Figure 2).

CRT values at three and 12 months were significantly lower than at baseline (P = 0.005 & P < 0.001, respectively). CRT significantly decreased between three and 12 months (P = 0.036). 

Table 2 summarized the results of comparison between the ranibizumab and aflibercept groups.

**Table 2:** Comparisons of characteristics between patients treated using aflibercept (aflibercept group) and those treated using ranibizumab (ranibizumab group). Data presented as mean ± standard deviation where applicable. BCVA: best-corrected visual acuity; ETDRS: Early Treatment Diabetic Retinopathy Study. *Statistical analysis performed using the independent samples t-test. ^†^Statistical analysis performed using the chi-square test. ^‡^Statistical analysis performed using the Mann-Whitney U test.

Baseline			
	Aflibercept (n = 44)	Ranibizumab (n = 33)	P-value
Age, years	81.3 ± 7.3	78.8 ± 7.4	0.173*
Sex, no. (%)			0.717^†^
Men	15 (38.5%)	12 (42.8%)	
Women	24 (61.5%)	16 (57.2%)	
Type of neovascularization, no (%)			0.474^†^
Classic	15 (34%)	9 (27.3%)	
Occult	15 (34%)	9 (27.3%)	
Mixed	14 (32%)	15 (45.4%)	
Size of lesion, DA	2.6 ± 1.3	2.7 ± 1.4	0.849^*^
Surface (%)	60% ± 7%	60% ± 7%	0.843^*^
Central retinal thickness, µm	414.3 ± 97.8	384.9 ± 97.9	0.158^‡^
BCVA (ETDRS)	64.2 ± 9.3	67.9 ± 8.6	0.101^‡^
After treatment at 12 months			
Central retinal thickness, µm	345.4 ± 73.7	398.9 ± 127.2	0.073^‡^
BCVA (ETDRS)	69.1 ± 9.4	68.1 ± 12.2	0.917^‡^
Number of injections	6.7 ± 1	4.91 ± 1.5	<0.001^‡^

From factors listed above, only number of injections differed significantly. In the ranibizumab group, patients received 4.9 ± 1.5 injections. In the aflibercept this was significantly higher 6.7 ± 1 (P < 0.001). In the ranibizumab group, 11 (33%) eyes received < 5 injections. There were no statistically significant changes between baseline BCVA, CRT and 12 months BCVA, CRT in eyes, which received < 5 injections (P = 0.108 & P = 0.929 & P = 0.077, respectively). 22 eyes (66%) received $\geq$5 injections in the ranibizumab group. There were no statistically significant changes between baseline BCVA, CRT and 12 months BCVA, CRT for these eyes (P = 0.842 & P = 0.702 & P = 0.136, respectively). In the aflibercept, 9 (20%) eyes, received < 7 injections. There were no significant changes between baseline BCVA & CRT and 12 months BCVA & CRT for these eyes (P = 0.213 & P = 0.133 & P = 0.548, respectively). 35 eyes (80%) received $\geq$ 7 injections of the aflibercept. In this group, baseline BCVA (63.4 ± 9.1) significantly improved after 12 months of treatment (68.9 ± 10.1; P < 0.001). There was significant decrease in CRT (P < 0.001), from 422.8 ± 102.5 at baseline to 341.2 ± 69.9 at 12 months. 

The results of comparison of baseline characteristics between the good visual outcome and poor visual outcome groups were presented in Table 3 (aflibercept group) and Table 4 (ranibizumab group).

**Table 3:** Comparisons of baseline characteristics on the basis of the best-corrected visual acuity at 12 months (≥ 20/50 or < 20/50) in patients treated with aflibercept. Data presented as mean ± standard deviation where applicable. BCVA: best-corrected visual acuity; ETDRS: Early Treatment Diabetic Retinopathy Study. ^†^Statistical analysis performed using the chi-square test. ^‡^Statistical analysis performed using the Mann-Whitney U test.

Characteristics	BCVA $\geq$ 20/50 (n = 30)	BCVA <20/50 (n = 9)	P-value
Age, years	82.1 ± 7.2	78.9 ± 7.7	0.341^‡^
Sex, no. (%)			0.25^†^
Male	13 (43.3%)	2 (22.2%)	
Female	17 (56.7%)	7 (77.8%)	
Type of neovascularization, no. (%)			0.26^†^
Classic	11 (31.4%)	4 (44.4%)	
Occult	14 (40%)	1 (11.2%)	
Mixed	10 (28.6%)	4 (44.4%)	
Size of lesion, DA	2.5 ± 1.2	3 ± 1.7	0.554^‡^
Surface (%)	60% ± 7%	61% ± 5%	0.470^‡^
Central retinal thickness, µm	423.6 ± 100.8	374.3 ± 77.7	0.20^‡^
BCVA (ETDRS), at diagnosis	66.3 ± 8.1	56.7 ± 10.4	0.021^‡^
Number of injections	6.7 ± 1	6.9 ± 0.8	0.609^‡^

**Table 4:** Comparisons of baseline characteristics on the basis of the best-corrected visual acuity at 12 months (≥ 20/50 or < 20/50) in patients treated with ranibizumab. Data presented as mean ± standard deviation where applicable. BCVA: best-corrected visual acuity; ETDRS: Early Treatment Diabetic Retinopathy Study. ^†^Statistical analysis performed using the chi-square test. ^‡^Statistical analysis performed using the Mann-Whitney U test.

Characteristics	BCVA ≥20/50 (n = 21)	BCVA <20/50 (n = 7)	P-value
Age, years	78.2 ± 7.4	80.7 ± 7.9	0.447^‡^
Sex, no. (%)			0.077^†^
Male	11 (52.4%)	1 (14.3%)	
Female	10 (47.6%)	6 (85.7%)	
Type of neovascularization, no. (%)			<.001^†^
Classic	5 (20%)	4 (50%)	
Occult	15 (60%)	0 (0%)	
Mixed	5 (20%)	4 (50%)	
Size of lesion, DA	2.4 ± 1	3.6 ± 1.9	0.129^‡^
Surface (%)	60% ± 7%	60% ± 8%	0.989^‡^
Central retinal thickness, µm	374.2 ± 76.5	418.6 ± 148.6	0.721^‡^
BCVA (ETDRS), at diagnosis	70.2 ± 7.6	60.6 ± 7.9	0.007^‡^
Number of injections	4.9 ± 1.6	5.0 ± 1.2	1.000^‡^

In the ranibizumab group, the good visual outcome group contained 21 (75%) eyes and the poor visual outcome group 7 (25%). The BCVA at diagnosis was significantly better (P = 0.007) in the good visual outcome group (70.2 ± 7.6) than in the poor visual outcome group (60.6 ± 7.9). In addition, the prevalence of type 1 neovascularization was significantly greater in the good visual outcome group (60%), than in the poor visual outcome group (0%; P < 0.001). In the aflibercept group, the good visual outcome group contained 30 (77%) eyes and the poor visual outcome group contained 9 (23%) eyes. The BCVA at diagnosis was significantly better (P = 0.021) in the good visual outcome group (66.3 ± 8.1) than in the poor visual outcome group (56.7 ± 10.3).

## Discussion

In the present study, one initial injection of ranibizumab and then PRN regimen resulted in stabilization of neovascular AMD progression. The 12-month outcomes of treatment with ranibizumab were different than revealed in other studies [8-10]. Retrospective, observational studies [8-10] reported both significant improvement in BCVA and reduction in CRT at 12 months. Eyes treated with $\geq$ 5 number of injections had similar results as eyes, that received < 5 injections. Observed outcomes could be associated with administration of ranibizumab to eyes with better prognosis at baseline. This might result in lower BCVA improvement and CRT reduction at 12 months. In addition, these eyes might receive less injections. According to recommendations of the drug program [6]. ranibizumab was administered in PRN scheme. None of the analyzed patients received initial 3 monthly injections. This could also explain different outcomes than in cited studies. Finally, analyzed cohort was older than in cited retrospective observational studies, what potentially could affect the treatment outcomes. 

Aflibercept was found to be effective in treating patients with the neovascular AMD. There was statistically significant visual acuity improvement and CRT reduction. At 12 months, patients gained 4.9 ± 8.5 ETDRS letters and CRT was reduced by 72 ± 90 µm, from the baseline. Similar results were revealed in another retrospective, observational studies with 12-months follow-up [11-15]. Less than seven injections resulted in non-significant changes in BCVA and CRT. This coincides with other authors findings. Treatment-naïve wet AMD eyes receiving fewer than seven intravitreal aflibercept injections in the first year of treatment had worse visual outcomes [16-18].

In the present study, good baseline BCVA was associated with visual acuity of 20/50 or better at 12 months in both the ranibizumab and aflibercept groups. Previous studies showed that type 1 neovascularization was associated with a better long-term visual outcome than other subtypes of neovascularization [15,19]. In ranibizumab group, the proportion of cases involving type 1 neovascularization was greater in the good visual outcome than in the poor visual outcome group. This trend was not revealed in the aflibercept group. The difference in the ranibizumab group, could be potentially due to the small sample size. However, this was statistically significant. 

The main limitations of this study were small number of patients and unclear allocation criteria. Future prospective investigations with clearly defined selection criteria would potentially bring different results. In addition, greater patient cohort would ensure treatment outcomes more similar to those reported in cited observational studies.

## Conclusions

The authors aimed to evaluate the effectiveness of Polish anti-VEGF therapeutic program in the setting of regional hospital. Three monthly intravitreal injections of aflibercept (2.0 mg/0.05 mL) and then every two months was an effective treatment regimen of naïve wet AMD eyes. This resulted in significant BCVA improvement and CRT reduction at 12 months of therapy. One initial monthly injection of ranibizumab (0.5 mg/0.05 mL) and then PRN regimen resulted in stabilization of the disease progression, for 12 months follow-up period in naïve wet AMD eyes. There was no significant difference in BCVA between ranibizumab and aflibercept at 12 months of therapy. The main limitations of this study were its retrospective, single-center nature and unclear allocation criteria. There were a relatively small number of patients and short follow-up period. Future prospective investigation, with clearly established allocation criteria and a greater number of patients should ensure comparable results to these reported in other observational studies. 
